# Genetic characterization of G12P[6] and G12P[8] rotavirus strains collected in six African countries between 2010 and 2014

**DOI:** 10.1186/s12879-020-05745-6

**Published:** 2021-01-22

**Authors:** Kebareng G. Rakau, Martin M. Nyaga, Maemu P. Gededzha, Jason M. Mwenda, M. Jeffrey Mphahlele, L. Mapaseka Seheri, A. Duncan Steele

**Affiliations:** 1 Diarrhoeal Pathogens Research Unit, Department of Virology, WHO AFRO Rotavirus Regional Reference Laboratory, Sefako Makgatho Health Sciences University, Pretoria, South Africa; 2 Next Generation Sequencing Unit and Department of Medical Microbiology and Virology, Faculty of Health Sciences, University of the Free State, Bloemfontein, South Africa; 3 National Health Laboratory Service, Department of Molecular Medicine and Haematology, Charlotte Maxeke Johannesburg Academic Hospital, Johannesburg, South Africa; 4 African Rotavirus Surveillance Network, Immunization, Vaccines and Development Cluster, WHO African Regional Office, Brazzaville, Congo; 5 South African Medical Research Council, Soutpansberg Road, Pretoria, South Africa; 6 Present address: Enteric and Diarrheal Diseases, Global Health, Bill & Melinda Gates Foundation, Seattle, WA USA

**Keywords:** Rotavirus strains, Africa, G12, P[8], P[6]

## Abstract

**Background:**

G12 rotaviruses were first observed in sub-Saharan Africa in 2004 and since then have continued to emerge and spread across the continent and are reported as a significant human rotavirus genotype in several African countries, both prior to and after rotavirus vaccine introduction. This study investigated the genetic variability of 15 G12 rotavirus strains associated with either P[6] or P[8] identified between 2010 and 2014 from Ethiopia, Kenya, Rwanda, Tanzania, Togo and Zambia.

**Methods:**

The investigation was carried out by comparing partial VP7 and partial VP4 sequences of the African G12P[6] and G12P[8] strains with the available GenBank sequences and exploring the recognized neutralization epitopes of these strains. Additionally, Bayesian evolutionary analysis was carried out using Markov Chain Monte Carlo (MCMC) implemented in BEAST to estimate the time to the most recent ancestor and evolutionary rate for these G12 rotavirus strains.

**Results:**

The findings suggested that the VP7 and VP4 nucleotide and amino acid sequences of the G12 strains circulating in African countries are closely related, irrespective of country of origin and year of detection, with the exception of the Ethiopian strains that clustered distinctly. Neutralization epitope analysis revealed that rotavirus VP4 P[8] genes associated with G12 had amino acid sequences similar to those reported globally including the vaccine strains in RotaTeq and Rotarix. The estimated evolutionary rate of the G12 strains was 1.016 × 10^− 3^ substitutions/site/year and was comparable to what has been previously reported. Three sub-clusters formed within the current circulating lineage III shows the diversification of G12 from three independent ancestries within a similar time frame in the late 1990s.

**Conclusions:**

At present it appears to be unlikely that widespread vaccine use has driven the molecular evolution and sustainability of G12 strains in Africa. Continuous monitoring of rotavirus genotypes is recommended to assess the long-term impact of rotavirus vaccination on the dynamic nature of rotavirus evolution on the continent.

**Supplementary Information:**

The online version contains supplementary material available at 10.1186/s12879-020-05745-6.

## Background

Diarrhoeal disease is a major cause of death in infants and young children below the age of 5 years and rotavirus is the most significant pathogen associated with that mortality [1]. Rotavirus is estimated to cause 122,232–215,757 Under-5 deaths annually [2, 3]. Furthermore, it has been estimated that diarrhoeal diseases are significantly more severe in immunocompromised children, especially those infected with HIV which is relevant to sub-Saharan Africa [4]. Recent estimates showed that the introduction of rotavirus vaccines globally has resulted in a relative reduction of 59% of rotavirus hospitalizations and 36% of all cause acute gastroenteritis hospitalizations, respectively [5]. Before the introduction of rotavirus vaccines in many African countries, it was estimated that almost 40% of all diarrhoeal cases on the continent were due to rotavirus infection [4]. The introduction of rotavirus vaccines into 29 sub-Saharan African countries before 2016, resulted in a reduction of approximately 21,000 deaths and 135,000 hospitalizations in 2016 alone [6], highlighting the major impact that rotavirus vaccines have had on rotavirus diarrhoea.

Rotaviruses are double-stranded RNA (dsRNA) viruses, which belong to the family *Reoviridae* [7]. The viral genome comprises of eleven segments which code for six structural viral proteins (VP; labelled VP1-VP4, VP6-VP7) and six non-structural proteins (NSP; NSP1-NSP6). Two of the structural proteins (VP7 and VP4) form the outer capsid of the virus, which are used in the binomial classification of rotavirus strains into G (for the VP7 glycoprotein) and P (for the VP4 protease-sensitive) types, respectively. According to the Rotavirus Classification Working Group of the International Committee on Taxonomy of Viruses (ICTV), there are 36 G and 51 P rotavirus types causing diarrhoea in humans, animals and avian species [7–9]. Of these, only six genotypes - G1P[8], G2P[4], G3P[8], G4P[8], G9P[8] and G12P[8] - are responsible for over 90% of rotavirus-related diarrhoea in humans globally [10–12].

On the African continent the dominant VP7 genotypes of rotavirus are G1, G2, G3, G4, G8, G9 and G12; G4 strains predominated in the 1980s and 1990s but have subsequently diminished dramatically [13]. The common VP4 genotypes of human rotaviruses circulating are P[8], P[6] and P[4] [14–16], with an unusually high prevalence of P[6] in Africa [16]. The emergence and rapid spread of G12 rotavirus strains has been widely observed globally [17]. A decade after the first report of G12 strains in the Philippines in 1987 [18], which was believed to be a zoonotic transmission to a child, the widespread circulation of this genotype was reported in South and North America, Asia and Europe [11, 18–21]. In sub-Saharan Africa, G12 strains initially emerged in combination with the VP4 P[6] genotype and were first reported in South Africa in 2004 during a hospital-based rotavirus surveillance study [22]. Subsequently, G12 rotavirus strains were reported in other African countries such as Malawi, Nigeria, Ghana, Cameroon, Kenya, Tanzania, Ethiopia, Zambia, Togo, and Zimbabwe [23–29].

Interestingly, genotype G12 strains were prevalent in Africa during the evaluation of the available rotavirus vaccines (i.e. Rotarix and RotaTeq), in large safety and efficacy studies [30, 31]. Neither of these vaccines contain the G12 VP7 genotype although both have a VP4 P[8] genotype. RotaTeq (Merck & Co., White River, Pennsylvania, USA) is a pentavalent bovine-human mono-reassortant vaccine containing 4 VP7 reassortants carrying the human G1 - G4 encoding genes and a VP4 reassortant carrying the human P[8] encoding gene, all on the genetic background of bovine rotavirus WI79 (G6 P[5]) strain [32]. Rotarix (GSK Biologicals, Rixensart, Belgium) is a human rotavirus strain bearing a G1P[8] genotype. The strain was isolated in 1989 [33].

Both rotavirus vaccines demonstrated homotypic and heterotypic protection against the common circulating strains in multiple studies in diverse geographies, including the circulating genotype G12 strain [34–38]. Furthermore, various post-marketing surveillance studies have reported that the vaccine confers heterotypic protection against novel strains carrying neither VP4 nor VP7 antigens found in the vaccines [39]. Nevertheless, there is concern in the scientific community about the issue of “vaccine-induced immune pressure” driving the emergence of novel strains that may evade vaccine protection [40]. As G12 rotavirus strains are the most recent to emerge and spread globally and are circulating in several African countries that have introduced the vaccine, and as information about the temporal genetic diversity of the circulating G12 strains in Africa is still limited, we sought to investigate the genetic variability of the two recognized neutralization antigens, VP7 and VP4, of G12 strains from across the continent. Thus, this study investigated the genetic variability of the gene segments 4 (encoding VP4) and 9 (encoding VP7) of G12 strains identified in several African countries and analysed the putative neutralization epitopes in an effort to provide insights on the evolutionary mechanisms and possible origins of the G12 strains in Africa.

## Methods

### Ethical approval

The University of Limpopo (MEDUNSA campus) (now called Sefako Makgatho Health Sciences University) Research & Ethics Committee approved the study (MREC/P/237/2014).

The diarrheal stool samples were collected as a routine diagnostic clinical specimen when the parents brought their child to a health facility for clinical management, requiring no written informed consent. As part of the World Health Organization (WHO) coordinated rotavirus surveillance network, the archived rotavirus-positive specimens, were anonymized and utilized for strain characterization under a Technical Service agreement and a Materials Transfer Agreement (MTA) to the WHO Regional Office for Africa (WHO AFRO) Regional Reference Laboratory based at Sefako Makgatho Health Services University. The WHO Research Ethics Review Committee granted an ‘exemption activity’, noting that the procedures involved in the study are part of routine hospital-based rotavirus surveillance.

### Sample collection

The stool samples were collected from children presenting with diarrhea during the 2010–2012 and 2014 rotavirus surveillance periods from six African countries (Ethiopia (ETH), Kenya (KEN), Rwanda (RWA), Tanzania (TZA), Togo (TGO), and Zambia (ZMB)). A standardised WHO generic protocol for hospital–based rotavirus surveillance was followed to recruit eligible children and collect the stool samples, as described elsewhere [14, 15]. The samples were available at the Diarrhoeal Pathogens Research Unit (DPRU), a WHO Rotavirus Regional Reference Laboratory for rotavirus strain characterization based at Sefako Makgatho Health Sciences University. The samples were stored at − 20 °C until retrieved for this analysis. Fifteen rotavirus strains previously recorded as G12 by conventional genotyping methods [15] were selected for further analysis in this study. Table 1 lists the characteristics of the 15 selected G12 strains analysed in this study.

**Table 1:** Demographics of the 15 G12P[8] and G12P[6] rotavirus genotypes

Country of isolation	Common name	Year of identification	G-P types	VP4,VP7 accession number	Rotavirus vaccine introduction
Ethiopia	MRC-DPRU2268	2011	G12P[8]	MK059445, MK059430	November 2013 Rotarix®
Ethiopia	MRC-DPRU2273	2011	G12P[8]	MK059448, MK059438	
Ethiopia	MRC-DPRU4959	2011	G12P[8]	MK059446, MK059432	
Ethiopia	MRC-DPRU5683	2014	G12P[8]	MK059442, MK059429	
Ethiopia	MRC-DPRU857	2012	G12P[6]	MK059443, MK059431	
Kenya	MRC-DPRU1367	2012	G12P[6]	MT995938, MK059435	July 2014 Rotarix®
Kenya	MRC-DPRU1369	2012	G12P[6]	MK059435, MK059436	
Kenya	MRC-DPRU1377	2012	G12P[6]	MK059449, MK059437	
Kenya	MRC-DPRU4288	2010	G12P[6]	MK059447, MK059433	
Rwanda	MRC-DPRU6219	2014	G12P[8]	MK059441, MK059439	May 2012 RotaTeq®
Tanzania	MRC-DPRU4540	2011	G12P[8]	MK059451, MK059439	January 2013 Rotarix®
Togo	MRC-DPRU2118	2011	G12P[8]	MK059452, MK059440	June 2014 Rotarix®
Zambia	MRC-DPRU1765	2012	G12P[6]	MK059444, MK059426	January 2012 Rotarix®
Zambia	MRC-DPRU2495	2011	G12P[6]	MT995937, MK059427	
Zambia	MRC-DPRU4165	2010	G12P[8]	MK059450, MK059434	

### Viral dsRNA extraction, VP4 and VP7 genotyping

Viral dsRNA was extracted using QIAamp® viral RNA extraction kit (Qiagen, Hilden, Germany) as per manufacturer’s instructions. The extracted dsRNA was subjected to reverse transcription polymerase chain reaction (RT-PCR) to amplify VP4 (partial VP8*) and VP7 genes using consensus primers sets Con2/Con3 and sBeg/End9, respectively [15, 22, 27]. Furthermore, to confirm the samples as G12 rotavirus strains, samples were genotyped using a cocktail of primers consisting of RVG9 and aBT1, aCT2, mG3, aDT4, aAT8v, mG9, mG10, newG12, representing G1, G2, G3, G4, G8, G9, G10 and G12 genotypes [37, 38]. The VP4 gene cocktail of primers which amplifies VP8* consisted of Con3 and 1 T-1D, 2 T-1, 3 T-1, 4 T-1 and 4943 representing human rotavirus genotypes P[8], P[4], P[6], P[11] and P[14] [27, 37]. The sequences of primers used in this study are shown in Supplementary Table 1. The PCR conditions were set out as described elsewhere [22, 37, 41, 42].

### Sanger sequencing

Amplicons were sequenced using the dideoxynucleotide termination Sanger sequencing method with ABI 3500XL sequencer. A region of VP7 and VP4 was sequenced using reverse and forward primers used for RT-PCR. The sequence chromatograms were edited using chromasPro version 1.49 beta resulting in 981 bp located at position 1–981 of the VP7 gene and approximately 731 bp located from position 97–827 of the VP4 gene fragments (www.technelysium.com.au/chromas.html).

### Sequence analysis

Sequencing data was then compared with available rotavirus sequences in the GenBank database using the NCBI-BLAST software (www.ncbi.nlm.nih.gov/BLAST/, USA). The VP7 and VP4 alignments were made using the MUSCLE algorithm implemented in MEGA 6 software [43, 44]. To expand the analysis, VP7 G12 and VP4 P[8] sequences from other African countries available in the GenBank were downloaded and included in the alignments. Once aligned, the DNA Model Test program implemented in MEGA version 6 was used to identify optimal evolutionary models that best fit sequence datasets. Using the Corrected Akaike Information Criterion (AICc) the following models; GTR + G (VP7), T92 + G (VP4 P[8]) and GTR + G + I (VP4 P[6]) were utilized. Using these models, maximum-likelihood trees were constructed using MEGA 6 with 1000 bootstrap replicates to estimate branch support. Nucleotide and amino acid sequence identities among strains were calculated for each gene based on distance matrices prepared using the p-distance algorithm in MEGA 6 software [43]. Dot conservation plots were constructed using BioEdit sequence alignment editor [45] identifying the variable and antigenic regions within the VP7 gene [46, 47] of the study strains with G12 reference strains belonging to the four G12 lineages (I-IV).

Simultaneously, P[8] VP4 sequences of the study strains were compared with P[8] of both the Rotarix and RotaTeq vaccine strains and other recent circulating strains; while the P[6] VP4 sequences were analysed by comparison with other globally circulating P[6] reference strains.

To estimate the rate of evolution (substitutions per site per year) and the time of the most recent common ancestor of the G12 genotype, 114 G12 VP7 sequences isolated between 1987 and 2019, representing the temporal span of these genotypes from the first G12 isolate to contemporary strains, and spanning global distribution of these strains, were retrieved from GenBank together with our study strains. To investigate the temporal signal of the sequences and to remove sequences that might be diverse, the G12 maximum likelihood phylogenetic tree was analysed in TempEST v1.5.3 [48], a tool that assesses the association of root-to-tip divergence and sampling of each sequence. Finally, Bayesian Markov chain Monte Carlo (MCMC) analysis was performed in BEAST v.1.6 software package, (http://beast.bio.ed.ac.uk). Several models with different priors were initially tested and compared using Bayes factor. Then the following Bayesian parameters were set out - GTR + G substitution model, uncorrelated exponential relaxed clock lognormal model [49] and coalescent Bayesian skyline tree prior [50]. This analysis was run four times at 50 million generations. The individual runs were combined with LogCombiner and Tracer v.1 (http://tree.bio.ed.ac.uk/software/tracer/) was used to view the results and effective sampling size (ESS) values of > 200 indicated sufficient sampling. Maximum clade credibility trees were annotated using TreeAnnotator v.1.6.2 and visualized in FigTree v.1.4.3 (http://tree.bio.ed.ac.uk/software/figtree/).

### Accession numbers

The partial VP7 and VP4 sequences have been made available on the NCBI GenBank database (Accession numbers: MK059426 - MK059453; MT995937-MT995938).

## Results

### VP7 genotype analysis

The nucleotide and amino acid sequences of 15 G12 rotavirus strains collected during 2010–2014 rotavirus seasons across Africa, were analysed and compared with the strains from the GenBank database. High nucleotide (96.8–99.9%) and amino acid (98.1–100%) sequence similarity was observed amongst the VP7 gene sequence of the G12 study strains as well as between the study strains and circulating global human G12 strains. One strain, MRC-DPRU6219 from Rwanda, shared 95.8–98.0% nucleotide and 97.2–98.7% amino acids identity with the other 14 study strains (Supplementary Table 2).

**Fig. 1:**
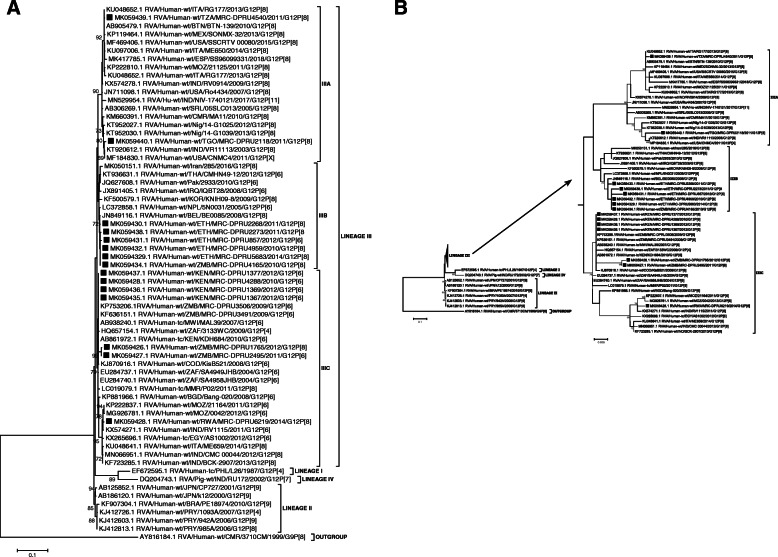
**a** Complete Maximum likelihood G12 tree illustrating the branching of the four G12 lineages and sub-lineages. **b** G12P[8] and G12P[6] VP7 maximum likelihood tree constructed from African G12 rotavirus nucleotide sequences from six countries and selected published human and porcine rotavirus reference strains. Bootstraps > 70 are shown on the branch length. Key: The African G12 strains are indicated in black and the countries abbreviated as follows: Ethiopia-ETH, Kenya-KEN, Rwanda-RWA, Tanzania-TZA, Togo-TGO and Zambia-ZMB

Phylogenetic analysis showed that the African rotavirus genotype G12 VP7 sequences clustered within lineage III, and sub-lineage III A-C (labelled for the purpose of discussion in this study) (Fig. 1). The two study strains, MRC-DPRU4540 (Tanzania) and MRC-DPRU2118 (Togo) in sub-lineage IIIA were closely related to globally circulating non-African G12 strains, and to Nigerians strains collected in 2012 and 2013, respectively. The G12 strains included in this study from Ethiopia (MRC-DPRU2268, MRC-DPRU857, MRC-DPRU2273, MRC-DPRU5683, MRC-DPRU4959 and MRC-DPRU4165) formed a monophyletic cluster within sub-lineage IIIB with reference strains from Nepal and Belgium. The study strains isolated from Kenya and Zambia clustered in sub-lineage IIIC and closely related to other African G12 strains. Interestingly most of these African strains all shared a G12P[6] genotypic constellation. Within the same IIIC sub-lineage a single Rwandan strain (MRC-DPRU6219) isolated in 2014 seemed distinct and clustered closer to strains isolated from Mozambique and India.

**Fig. 2:**
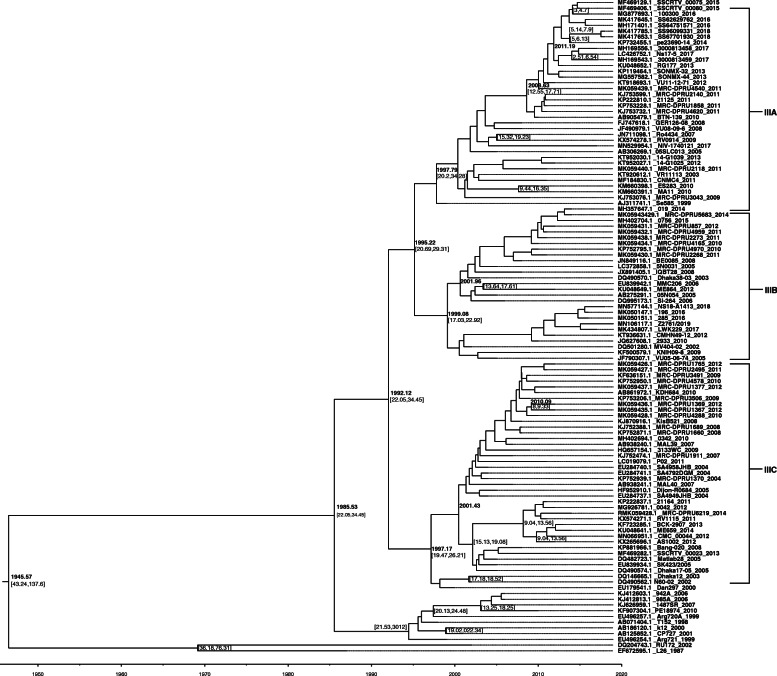
G12 Maximum Credibility Clade phylogenetic tree based on 114 G12P[8] and G12P[6] human and porcine rotavirus strains. For each G12 strain, the accession number, name and year are mentioned. The ages and height 95% HPD are shown on the nodes. Our study strains are indicated by a black arrow and the three sub-clusters are labelled

The temporal signal analysis of G12 maximum likelihood phylogenetic tree analysed in TempEST reported a correlation coefficient of 0.6213 and R-squared value of 0.386 which suggests a strong association and high effect size of the G12 sequences. The linear regression plot shows clustering of G12 sequences around 2010 (Supplementary Figure 1). Evolutionary analysis in BEAST generated ESS values that were > 200 for all parameters. The estimated evolutionary rate for G12 was 1.16 × 10^− 3^ nucleotide substitutions/site/year with 95% high posterior density interval (95%HPD) of 7.23 × 10^− 4^ to − 1.30 × 10^− 3^. The most recent common ancestor for lineage III, which consists of the current circulating strains, was dated back to 1992 (Fig. 2). Maximum clade credibility tree also displayed the diversification of lineage III strains into sub-clusters similar to the maximum likelihood phylogenetic tree. The three sub-clusters most common recent ancestors were estimated to 1997 for IIIA, 1999 for IIIB and 1997 for IIIC.

Sequence analysis within the nine variable regions (VR) and four antigenic regions (AR, A-C,F) of VP7 of the study strains were considerably conserved when compared to the first reported African G12 strain from South Africa (SA4958JHB) as well as representative strains belonging to the four lineages. The comparison of the amino acid showed differences mostly within the variable regions of the gene compared to the antigenic regions which carry the recognized antigen-specific epitopes (Table 2). Although the study strains were related to the first reported South African strain, at certain positions the study strains shared amino acids similar to the prototype (L26, lineage I) and porcine (RU172, lineage IV) G12 strains. For instance, amino acid substitution M44I observed in multiple study strains was similar to the L26 and RU172 strains.

**Table 2:**
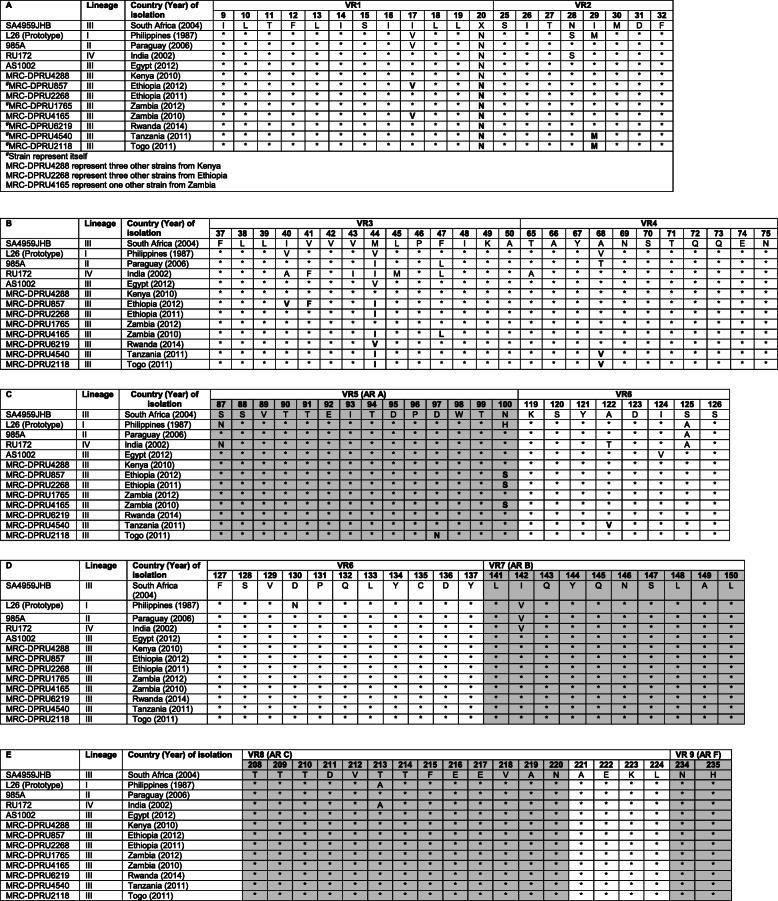
A-E: Comparison of the nine variable regions (VR) and four antigenic regions (AR) in VP7 (defined in references [46, 47]) of the 15 study strains with reference strains representing all four G12 lineages. Amino acid substitutions within the study strains are bolded, grey areas indicate antigenic regions and * indicates that the amino acid is conserved

Interestingly, in antigenic region A, strains from Ethiopia and Zambia had an N100S amino acid substitution. An alignment of G12 lineage III strains circulating globally identifies this substitution as found in strains from Nepal, Italy and Belgium but not from other African strains (data not shown). Certain amino acids were unique to strains belonging to specific lineages such as amino acid substitutions – N100H, D130N in the prototype strain (L26, lineage I), the I40A, T65A, A122T in the porcine strain (RU172, lineage IV) and A68T in the 985A strain (lineage II). Furthermore, notable amino acid substitutions A125S and V142I differentiated lineage III isolates from those in lineages I, II and IV. This substitution was seen in all global G12 strains belonging to lineage III.

### VP4 genotype analysis

**Fig. 3:**
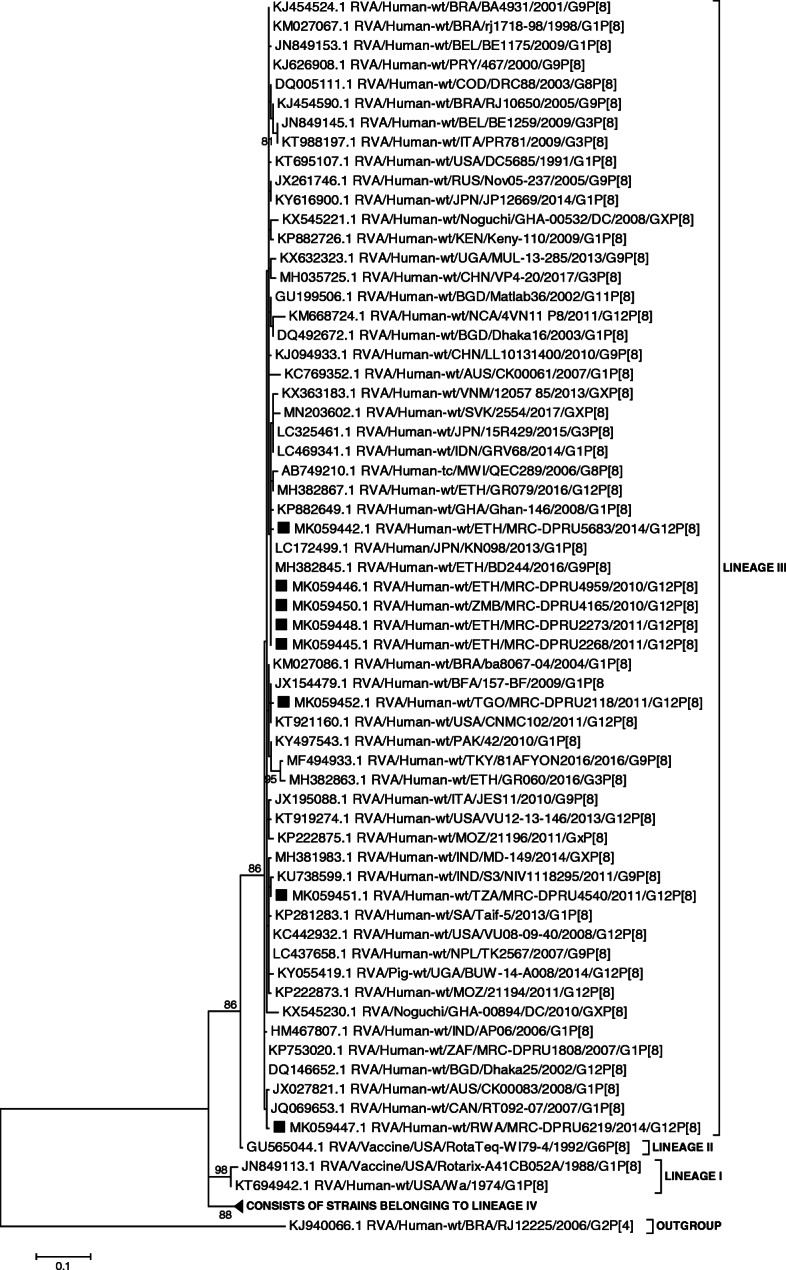
G12P[8] VP4 maximum likelihood tree constructed from African rotavirus nucleotide sequences from six countries and selected human rotavirus reference strains. Partial and complete sequences for reference strains were included in the analysis. Bootstraps > 70 are shown on the branch length. Key: The study sequences are indicated in black and the countries are abbreviated as follows: Ethiopia-ETH, Kenya-KEN, Rwanda-RWA, Tanzania-TZA, Togo-TGO and Zambia-ZMB

The partial VP8* gene sequence of VP4 (876 bp) was also analysed for the 15 study strains and compared to sequences in GenBank. Of the sequences analysed, both P[8] (*n* = 8) and P[6] (*n* = 7) strains were included. Sequence comparison showed that the G12P[8] strains shared 98.4–99.9% nucleotide and 98.1–100% amino acid similarity with most African P[8] strains available in the GenBank database. Also, they shared similar percentage similarity with each other (Supplementary Table 3). The P[8] rotavirus strains clustered in lineage III distantly from the Rotarix and RotaTeq P[8] vaccine components, which clustered in lineage I and lineage II, respectively (Fig. 3). Four of the five strains from Ethiopia formed their own monophyletic cluster as was also seen with their VP7 sequences. While MRC-DPRU5683 (Ethiopia), MRC-DPRU2118 (Togo), MRC-DPRU6219 (Rwanda) and MRC-DPRU4540 (Tanzania) scattered throughout the phylogenetic tree, clustering closer to strains from Hungary, USA, Australia and India, respectively.

The comparison of the eight strains bearing VP4 P[8] genotype with the VP4 P[8] gene included in the two vaccines, Rotarix and RotaTeq, and other strains representing different P[8] lineages, revealed that the strains are highly conserved with a few amino acid substitutions within the VP8* neutralizing antigenic epitopes. Within VP8* there are four defined neutralization epitopes, designated 8–1 to 8–4 [51] (Table 3). As shown in Table 3, the study strains had similar amino acid substitutions (S125N and S131R) with the VP4 of RotaTeq. This S131R substitution is common with other strains in lineages II to IV, but not with Rotarix which lies in lineage I. At positions 150 and 113, both the vaccine VP4 components were the same as the study strains (except for the monophyletic strains from Ethiopia which carried E150D and N113D substitutions). The S146G amino acid change was observed in all the study strains and the lineage III-IV reference strains, and distinct from Rotarix and RotaTeq. Finally, the study strains as well as the lineage III reference strain, had N195G amino acid change differentiating lineage III from lineages I (Rotarix), II (RotaTeq) and IV (MRC-DPRU2144).

**Table 3:** A-B Comparison of G12P[8] rotavirus strains with P[8] vaccine components of Rotarix and RotaTeq and other recent strains representing the P[8] lineages within the VP8* antigenic epitopes (defined in references [47, 50]). Amino acids substitutions within the studied strains are bolded and * indicates the amino acid is conserved

**A**	**P[8] Lineage**	**Country (Year) of isolation**	**8–1**										**8–2**		**8–3**					
			**100**	**146**	**148**	**150**	**188**	**190**	**192**	**193**	**194**	**195**	**180**	**183**	**113**	**114**	**115**	**116**	**125**	**131**
Rotarix® (Vaccine)	I	USA (1989)	D	S	Q	E	S	T	N	L	N	N	T	A	N	P	V	D	S	S
RotaTeq® (Vaccine)	II	USA (1992)	*	*	*	*	*	*	*	*	*	D	*	*	*	*	*	*	N	R
GR079	III	Ethiopia (2016)	*	G	*	D	*	*	*	*	*	G	*	*	D	*	*	*	N	R
MRC-DPRU2144	IV	South Africa (2004)	*	G	*	*	*	*	D	*	*	S	*	*	*	*	*	*	*	R
MRC-DPRU2268	III	Ethiopia (2011)	*	**G**	*	**D**	*	*	*	*	*	**G**	*	*	**D**	*	*	*	**N**	**R**
^#^MRC-DPRU6219	III	Rwanda (2014)	*	**G**	*	*	*	*	*	*	*	**G**	*	*	*	*	*	*	**N**	**R**
^#^MRC-DPRU 2118	III	Togo (2011)	*	**G**	*	*	*	*	*	*	*	**G**	*	*	*	*	*	*	**N**	**R**
**B**	**P[8] Lineage**	**Country (Year) of isolation**	**8–3**			**8–4**														
			**132**	**133**	**135**	**87**	**88**	**89**												
Rotarix® (Vaccine)	I	USA (1989)	N	D	V	N	T	N												
RotaTeq® (Vaccine)	II	USA (1992)	*	*	*	*	*	*												
GR079	III	Ethiopia (2016)	*	*	*	*	*	*												
MRC-DPRU2144	IV	South Africa (2004)	*	*	*	*	*	*												
MRC-DPRU2268	III	Ethiopia (2011)	*	*	*	*	*	*												
MRC-DPRU6219	III	Rwanda (2014)	*	*	*	*	*	*												
MRC-DPRU 2118	III	Togo (2011)	*	*	*	*	*	*												

#Strain represent itself

MRCDPRU2268 represent three other strains from Ethiopia, one from Tanzania and one from Zambia

**Fig. 4:**
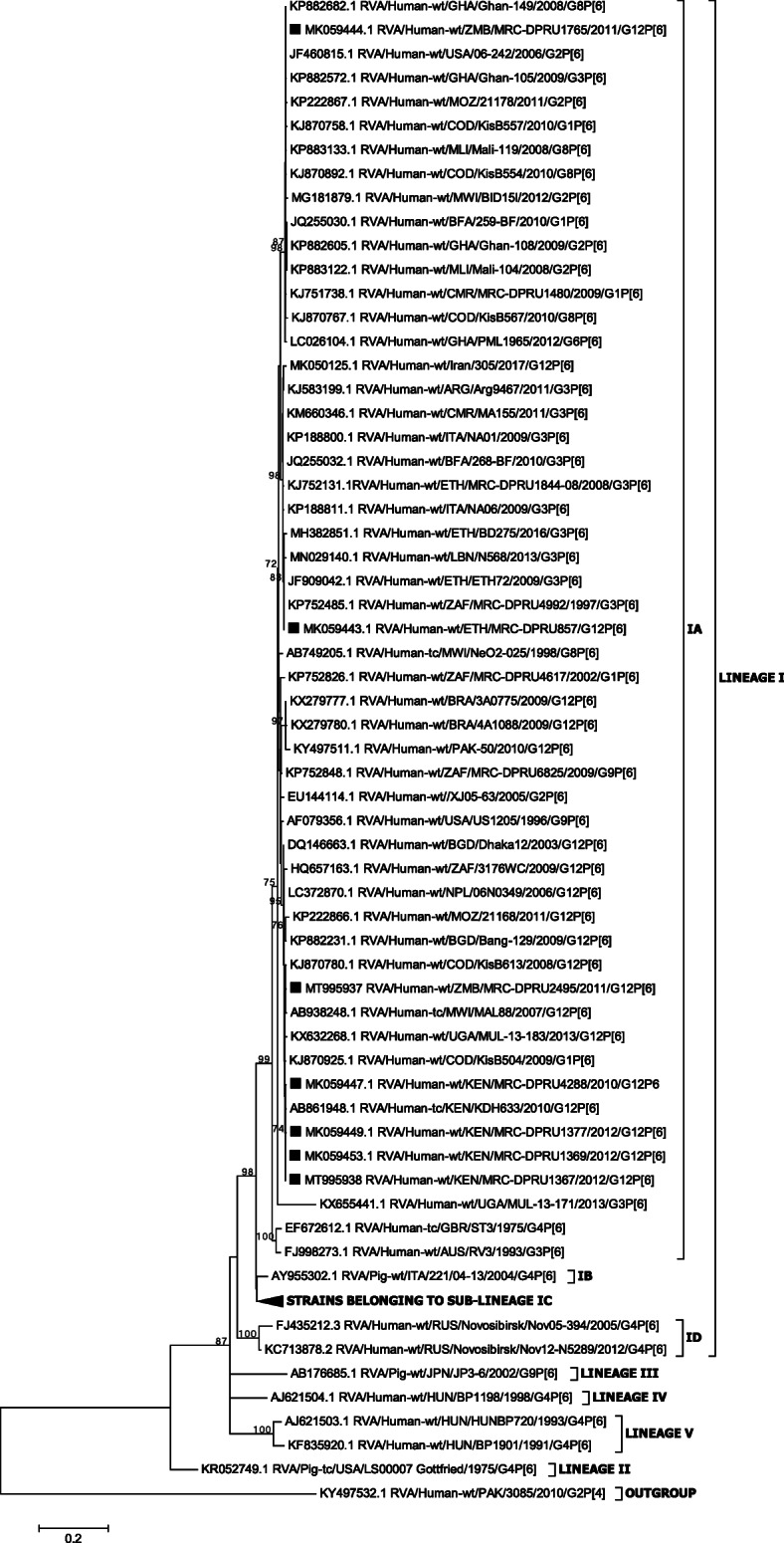
G12P[6] VP4 maximum likelihood tree constructed from African rotavirus nucleotide sequences from six countries and selected human rotavirus reference strains. Partial and complete sequences for reference strains were included in the analysis. Bootstraps > 70 are shown on the branch length. Key: The study sequences are indicated in black and the countries are abbreviated as follows: Ethiopia-ETH, Kenya-KEN, Rwanda-RWA, Tanzania-TZA, Togo-TGO and Zambia-ZMB

Similarly, the P[6] study strains shared 95.9–99.7% nucleotide and 97.1–100% amino acid similarity amongst themselves and with P[6] sequences available in the GenBank database. However, strain MRC-DPRU857 from Ethiopia shared fewer nucleotide and amino acid similarity with the study strains, 95.9–97.1% and 97.5–98.8% respectively (Supplementary Table 4). The P[6] study strains clustered in lineage Ia with other global strains and tended to cluster more closely with other African strains (Fig. 4). Amino acid conservation plot of the study strains with other P[6] strains representing the four lineages show that the study strains are conserved within lineage I into which the study strains clustered (Table 4). S146N was the only substitution detected in MRC-DPRU1369 strain isolated from Kenya, which was like reference strains representing lineage II-IV.

**Table 4:** A-B Comparison of G12P[6] strains with reference P[6] strains representing the four P[6] lineages within the VP8* antigenic regions [47, 50]. Amino acids substitutions within the studied strains are bolded and * indicates the amino acid is conserved

**A**	**P[6] Lineage**	**Country (Year) of isolation**	**8–1**										**8–2**		**8–3**					
			**100**	**146**	**148**	**150**	**188**	**190**	**192**	**193**	**194**	**195**	**180**	**183**	**113**	**114**	**115**	**116**	**125**	**131**
305	I	Iran (2017)	D	S	S	E	S	T	N	L	S	E	T	A	T	N	Q	S	T	E
Gottrified	II	USA (1975)	*	N	N	D	*	*	*	*	P	D	*	*	P	S	*	D	V	*
JP3–6	III	Japan	*	N	*	*	*	*	*	*	Y	D	*	*	V	S	*	*	*	*
B1198	IV	Hungary (1998)	*	N	*	*	*	*	*	*	*	*	*	*	*	*	*	*	*	*
MRC-DPRU1765	I	Zambia (2012)	*	*	*	*	*	*	*	*	*	*	*	*	*	*	*	*	*	*
MRC-DPRU1369	I	Kenya (2012)	*	**N**	*	*	*	*	*	*	*	*	*	*	*	*	*	*	*	*
^#^MRC-DPR857	I	Ethiopia (2012)	*	*	*	*	*	*	*	*	*	*	*	*	*	*	*	*	*	*
**B**	**P[6] Lineage**	**Country (Year) of isolation**	**8–3**			**8–4**														
			**132**	**133**	**135**	**87**	**88**	**89**												
305	I	Iran (2017)	N	N	N	T	N	Q												
Gottrified	II	USA (1975)	*	S	D	I	*	K												
JP3–6	III	Japan	*	*	*	*	*	*												
B1198	IV	Hungary (1998)	*	S	*	*	*	*												
MRC-DPRU1765	I	Zambia (2012)	*	*	*	*	*	*												
MRC-DPRU1369	I	Kenya (2012)	*	*	*	*	*	*												
MRC-DPR857	I	Ethiopia (2012)	*	*	*	*	*	*												

#Strain represents itself

MRC-DPRU1765 represent one other strain from Zambia

MRC-DPRU1369 represent three other strains from Kenya

## Discussion

This study analysed circulating G12P[6] and G12P[8] rotaviruses from several African countries collected during the period 2010–2014 and prior to widespread use of rotavirus vaccines on the continent. Genotype G12 strains, which emerged approximately two decades ago, have been reported to be the cause of severe dehydrating diarrhoea in vaccinated children in several countries, particularly in Latin America which started vaccination about six years prior to sub-Saharan Africa [52–54]. However, if one looks at a temporal association of the emergence of the G12 strains, it is associated with the global spread of these strains, rather than causally associated with wide-spread vaccine use. Nevertheless, with the introduction of rotavirus vaccine in 2012–2014 in many of the African countries included in this study, the opportunity existed to conduct an analysis of circulating G12P[6] and G12P[8] strains in several countries, just prior to and as vaccines were introduced and to evaluate whether these strains might become predominant due to evading the vaccine. Five of the six studied countries had introduced the Rotarix vaccine with the exception of Rwanda which initially introduced RotaTeq vaccine and switched to Rotarix in 2018.

Clearly, G12 strains do not share the VP7 G-specificity with vaccine strains; however, both licenced rotavirus vaccines (RotaTeq and Rotarix) have demonstrated clinical protection against heterotypic strains, including G12 strains. For instance, the phase III Rotarix clinical trial conducted in Malawi and South Africa showed cross protection against diverse rotavirus strains, including G12 with vaccine efficacy of 51.5% [37]. Similar results were observed with the RotaTeq vaccine study in three African countries [38]. Importantly, rotavirus vaccines have been shown to exercise protection via the immune responses to the VP4 neutralization antigens also [55], and the VP4 P[8] is shared between both vaccines and a proportion of the G12 strains evaluated, those with G12P[8]. Thus, understanding the genetic variability of both the VP4 and VP7 genes of the circulating G12 rotavirus strains should provide insights into the evolutionary relationships and potential biological advantages of these strains in Africa.

Phylogenetic analysis of G12 rotavirus strains globally, shows segregation of the strains into four lineages (I –IV). Lineage I is the prototype strain L26 identified in 1987 and which was not apparently biologically competitive in humans and did not spread; lineage II is the G12P[9] strains from Asia which appear to be a unique class of natural reassortants with a VP4 P[9]; and lineage IV includes the only porcine strain (G12P[7]) [17, 56, 57]. Lineage III strains, on the other hand, are the mostly contemporary G12 strains detected since the early-2000’s and which are now globally prevalent in most continents. This analysis confirms that the genotype G12 strains circulating in these six sub-Saharan African countries (Ethiopia, Kenya, Rwanda, Tanzania, Togo and Zambia) clustered in lineage III with strains circulating all over the world, showing the dominance and biological competitiveness of these strains, which have persisted over the last two decades in most continents [58–60]. The evolutionary rate of G12 genotype (1.016 × 10–3 nucleotide substitutions/site/year) observed in this study is well within the ranges that have been reported by several investigators [17, 61, 62]. The estimated time to the most recent ancestor of lineage III strains is 1992 which is similar to a previous estimate of 1995 [47] and the African strains - although scattered within the three sub-clusters - show their most recent ancestor to be from the late 1990s. This reflects the epidemiologic data, which reported the first isolation of G12 strains in the African continent in 2004. The observed diversification of our African strains in three sub-clusters is not due to their country of isolation, but more likely due to three different ancestral strains emerging at approximately the same time. This trend can be applied to globally circulating G12 strains belonging to lineage III [62].

Evidence of genetic variation was observed amongst the four G12 lineages in this study. Amino acid substitution S25N (VR2), N87S (antigenic region A) and A213T (antigenic region C) in lineages II & III, segregate between the prototype lineage I detected in 1987 and the porcine lineage IV. The lineages were further characterised by the amino acid substitutions A125S in VR6 and V142I in antigenic region B detected only in the current circulating lineage III strains. This latter change from Valine to Isoleucine, where the amino acids share similar chemical properties, might not impose a conformational change to the VP7 protein. However, the A125S substitution, in which Alanine acquired a hydroxyl group to change to Serine over the period of early 2000s to late 2000s could influence the capsid structure. The mechanism of rotaviruses mutating to advance epidemiological spread was observed with recent G2 rotavirus strains belonging to lineage IVa that spread globally. All these strains exhibited an amino acid substitution D96N which seemed to confer survival advantage to these lineage IVa G2 rotavirus strains [63]. It needs to be investigated further whether the A125S amino acid substitution observed in lineage III G12 strains has contributed to its competitiveness and spread. The amino acid substitutions and phylogenetic clustering of the study strains away from the porcine lineage IV, indicates that they are not genetically related although animal-human rotavirus transmission is often reported in the African continent.

Amino acids changes within the antigenic regions of VP7 can result in alteration to the antigenicity of the virus and potentially enhance immunity [47]. It has been shown that the antibodies targeting neutralization epitopes stabilize the capsid and prevent uncoating of the virus which is required for viral replication [64]. Zeller and colleagues proposed that differences in the neutralizing epitopes in VP4 could undermine the vaccines effectiveness [47]. If the vaccine efficacy is mediated through the VP4 antigen, then considering these mutations may provide further insight. The study strains had similar amino acids in most of the antigenic epitopes to the VP4 P[8] gene of RotaTeq, with some differences to Rotarix, which is the preferred vaccine in most African countries. The major amino acid substitution is in position 131, in which Rotarix had a Serine and RotaTeq and study strains had an Arginine.

The G12 rotaviruses appear to have emerged irrespective of the use of rotavirus vaccines and continue circulating in countries that have not introduced the vaccines, indicating the natural circulation and competitiveness of these human viral strains. For example, rotavirus vaccines were introduced in the six countries included in this study between 2012 and 2014 and the G12 strains analysed in this study were collected between 2010 and 2014. To substantiate further, various studies from Ethiopia have reported G12 rotaviruses as a dominant strain both pre- and post-vaccine introduction [65].

It is therefore not possible to conclude that the prevalence of G12 strains was affected by vaccine introduction. Possibly, assessing the G12 strains that have emerged in Latin America and Africa at different stages after rotavirus vaccine introduction might shed light on the evolutionary pressure exerted by the vaccines.

## Conclusion

The study findings suggest that the novel G12 strains circulating in African countries are highly similar at the nucleotide and amino acid level, irrespective of geographical distribution and year of detection. The African G12P[8] and G12P[6] rotavirus strains belonging to lineage III circulating in these countries are not unique and are the same as the globally circulating rotavirus G12 strains and there is no evidence of molecular evolutionary pressure from widespread vaccine use. The antigenic epitopes display limited diversity to each other and other global strains, including to the two rotavirus vaccines (RotaTeq and Rotarix), indicating that this is unlikely to be associated with sustained circulation over time in Africa. The G12 strains diversified into three sub-clusters with ancestries estimated to the late 1990s and are evolving at 1.016 × 10^− 3^ nucleotide substitutions/site/year sustaining this strain overtime. However, as new rotavirus vaccines which do not carry the common human rotavirus VP4 genotype (P[8]), such as RotaVac and RotaSIIL from India [66, 67] have been pre-qualified by WHO and are introduced in selected African countries, it will be imperative to continue genotypic surveillance to identify and monitor emerging strains.

## Supplementary Information

**Additional file 1.**

## Data Availability

The partial VP7 and VP4 sequences have been made available on the NCBI GenBank database (Accession numbers: MK059426 - MK059453, MT995937-MT995938).

## Abbreviations

AFRO: African Regional Office of WHO
AR: Antigenic regions
DPRU: Diarrheal Pathogens Research Unit
dsRNA: Double stranded Ribo-Nucleic Acid
ETH: Ethiopia
GSK: GlaxoSmith Kline
HIV: Human immunodeficiency virus
HPD: High posterior density
ICTV: International Committee for Taxonomy of Viruses
KEN: Kenya
MCMC: Markov Chain Monte Carlo
MTA: Materials Transfer Agreement
MREC: MEDUNSA Research and Ethics Committee
NSP: Non-structural protein
PCR: Polymerase chain reaction
RT-PCR: Reverse transcriptase polymerase chain reaction
RWA: Rwanda
TZA: Tanzania
TGO: Togo
VR: Variable regions
VP4: Viral protein 4
VP7: Viral protein 7
VP8*: Viral protein 8* where the * denotes a cleavage product of VP4
WHO: World Health Organization
