# Supplementary material for: Genetic characterization of G12P[6] and G12P[8] rotavirus strains collected in six African countries between 2010 and 2014
Source: BMC Infect Dis. 2021 Jan 22;21:107. doi: 10.1186/s12879-020-05745-6 (PMC7821174; doi:10.1186/s12879-020-05745-6)
Supplement: Supplementary file 1 — Additional file 1. [file 12879_2020_5745_MOESM1_ESM.docx]

Supplementary Table 1: The primers, primer sequences and fragment sizes to determine the VP4 and VP7 genotypes

| **REVERSE TRANSCRIPTION PCR PRIMERS** **(28-29)** | | |
| --- | --- | --- |
| VP4 (868 bp PCR fragment) | Con2 | ATTTCGGACCATTTATAACC |
|  | Con3 | TGGCTTCGCTCATTTATATAGACA |
| VP7 (1067 bp PCR fragment) | sBeg | GGCTTTAAAAGAGAGAATTTC |
|  | End9 | GGTCACATCATACAATTCTAATCTAAG |
| **GENOTYPING PRIMERS (28-31)** | | |
| P[8] (345 bp) | 1T-1D | TCTACTGGRTTRACNTGC |
| P[4] (483 bp) | 2T-1 | CTATTGTT GAGGTTAGAGTC |
| P[6] (267 bp) | 3T-1 | TGTTGATTAGTTGGATTCAA |
| P[14] (543 bp) | 4943 | GGTGTAGT CCTGCGTA |
| G1 (749 bp) | aBT1 | CAAGTACTCAAATCAATGATGG |
| G2 (652 bp) | aCT2 | CAATGATATTAACACATTTTCTGTG |
| G3 (812 bp) | mG3 | ACGAACTCAACACGAGAGG |
| G4 (583 bp) | aDT4 | CGTTTCTGGTGAGGAGTTG |
| G8 (885 bp) | aAT8v | GTCACACCATTTGTAAAYTCAC |
| G9 (305 bp) | mG9 | CTTGATGTGACTAYAAATAC |
| G10 (396 bp) | mG10 | ATGTCAGACTACARATACTGG |
| G12 (558 bp) | newG12 | GGTTATGTAATCCGATGGCG |
| *Key :R- either A/G N- either A/G/C/T Y- either C/T* | | |

Supplementary Table 2: VP7 Nucleotide and amino acid p-distance % similarity shared between studied G12P[6] and G12P[8] strains

Supplementary Table 3: VP4 Nucleotide and amino acid p-distance % similarity shared between studied G12P[8] strains

Supplementary Table 4: VP4 Nucleotide and amino acid p-distance % similarity shared between studied G12P[6] strains

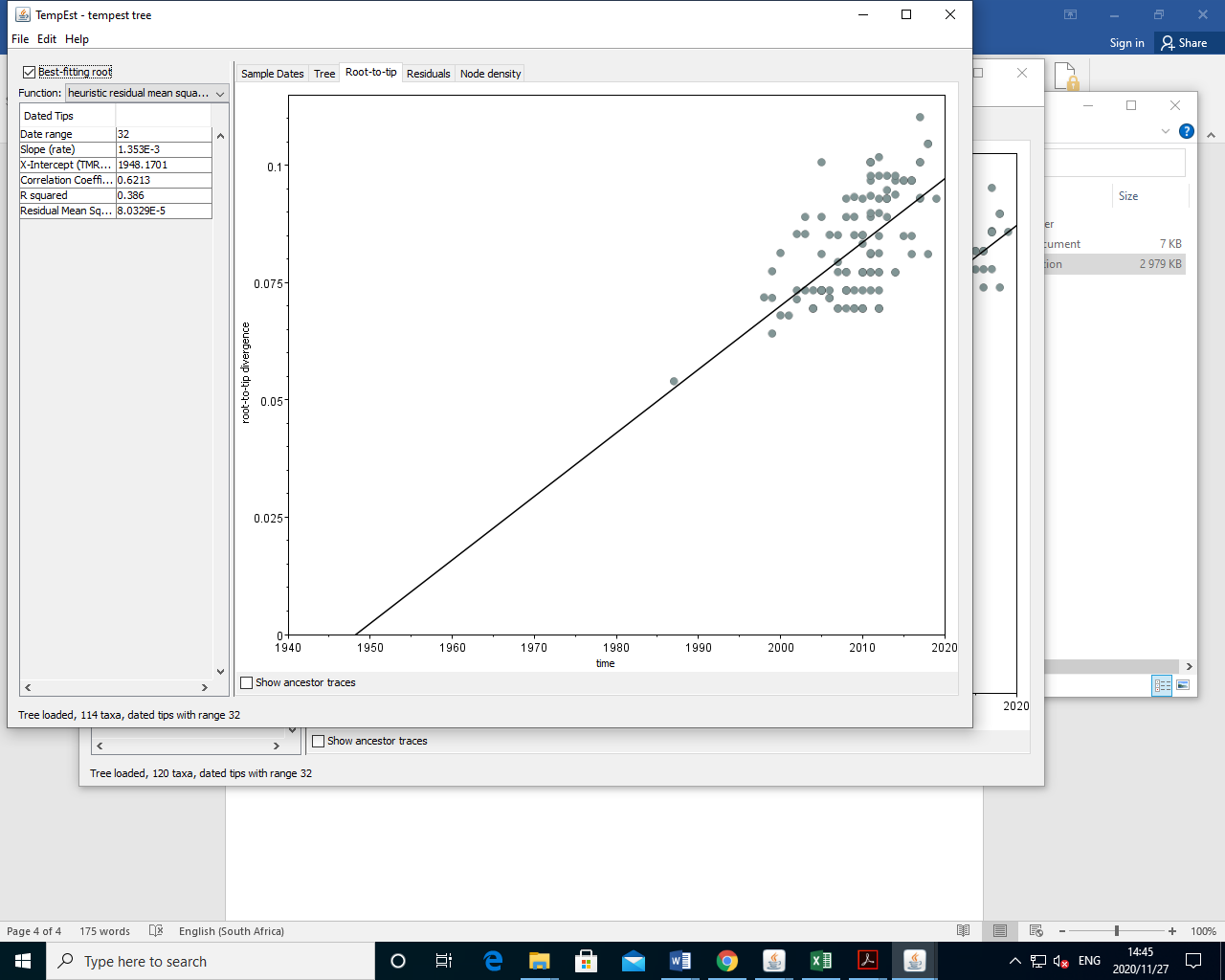


Supplementary Figure 1: Root-to-tip linear regression analysis of 114 global G12 strains including 51 African strains.
